# Role of Multifocal Visually Evoked Potential as a Biomarker of Demyelination, Spontaneous Remyelination, and Myelin Repair in Multiple Sclerosis

**DOI:** 10.3389/fnins.2021.725187

**Published:** 2021-10-29

**Authors:** Alexandr Klistorner, Stuart L. Graham

**Affiliations:** ^1^Department of Ophthalmology, The University of Sydney, Darlington, NSW, Australia; ^2^Department of Ophthalmology, Macquarie University, Sydney, NSW, Australia

**Keywords:** multiple sclerosis, demyelination, remyelination, visual evoked cortical potentials, clinical trial

## Abstract

Multiple sclerosis (MS) is a complex disease of the central nervous system (CNS), characterized by inflammation, demyelination, neuro-axonal loss, and gliosis. Inflammatory demyelinating lesions are a hallmark of the disease. Spontaneous remyelination, however, is often incomplete and strategies that promote remyelination are needed. As a result, accurate and sensitive *in vivo* measures of remyelination are necessary. The visual pathway provides a unique opportunity for *in vivo* assessment of myelin damage and repair in the MS-affected brain since it is highly susceptible to damage in MS and is a very frequent site of MS lesions. The visually evoked potential (VEP), an event-related potential generated by the striate cortex in response to visual stimulation, is uniquely placed to serve as a biomarker of the myelination along the visual pathway. The multifocal VEP (mfVEP) represents a most recent addition to the array of VEP stimulations. This article provides a current view on the role of mfVEP as a biomarker of demyelination, spontaneous remyelination, and myelin repair in MS.

Multiple sclerosis (MS) is a complex disease of the central nervous system (CNS), characterized by inflammation, demyelination, neuro-axonal loss, and gliosis. Inflammatory demyelinating lesions are a hallmark of the disease. The acute stage of lesion formation initially results in a complete block of conduction (and associated functional deficit) along the axons affected by the inflammation. Axonal conduction (and function), however, typically recovers within a few weeks, during which inflammation subsides, ion channels are reconstructed and conduction in surviving but demyelinated axons resumes, although often in a slower, continuous mode (Smith and Waxman, 2005). This restoration of conduction along the demyelinated axons is due to appearance of more widely distributed sodium channels that are diffusely deployed along demyelinated axolemma (Felts et al., 1997; Waxman, 2005).

Permanent demyelination, however, may contribute to accelerated degeneration of surviving axons by rendering them vulnerable to physiological stress (Kornek et al., 2000; Bruck et al., 2003). Chronic demyelination increases the energy demands of axonal conduction, ultimately compromising axoplasmic adenosine triphosphate (ATP) production, leading to an ionic imbalance and Ca^2+^-mediated axonal degeneration (Correale et al., 2017). In addition, lack of trophic support from myelin or oligodendrocytes and disruption of normal axon–myelin interactions may result in degeneration of chronically demyelinated axons (Trapp et al., 1999; Peterson and Fujinami, 2007).

While spontaneous remyelination was first described in MS in 1965 (Périer and Grégoire, 1965) and is now believed to be an early and frequent phenomenon occurring in MS (Raine and Wu, 1993; Patrikios et al., 2006), it is often incomplete (Prineas et al., 1989; Bramow et al., 2010) and strategies that promote remyelination are needed. A number of approaches to promote myelin repair have made significant progress in experimental models (Suhs et al., 2012) and it is now emerging as a new target for neuroprotective strategies, making its way into human clinical trials (see Cunniffe and Coles, 2019; Lubetzki et al., 2020 for review). Therefore, accurate and sensitive *in vivo* measures that can assess and verify the therapeutic and biological efficacy of putative remyelinating treatments are necessary in order for a transition to clinical therapy.

A number of imaging techniques have been suggested as potential surrogate biomarkers of myelin damage and repair in MS brain. There are several recent reviews examining the potential use of various imaging biomarkers in remyelination trials (Barkhof et al., 2009; Mallik et al., 2014; Oh et al., 2019). In this review, however, we concentrate on electrophysiological assessment of de/remyelination in MS patients.

The visual pathway provides a unique opportunity for *in vivo* assessment of myelin damage and repair in the MS-affected brain. Firstly, the visual system is highly susceptible to damage in MS and is a very frequent site of MS lesions. Optic neuritis (ON) is the presenting symptom of MS in approximately 20% of MS patients and evidence of previous ON is typically detected in half of the relapsing-remitting (RR) MS population, while optic radiation (OR) lesions are seen in about two-thirds (Figure 1; Hornabrook et al., 1992; Jenkins et al., 2010; Klistorner et al., 2015).

**FIGURE 1:**
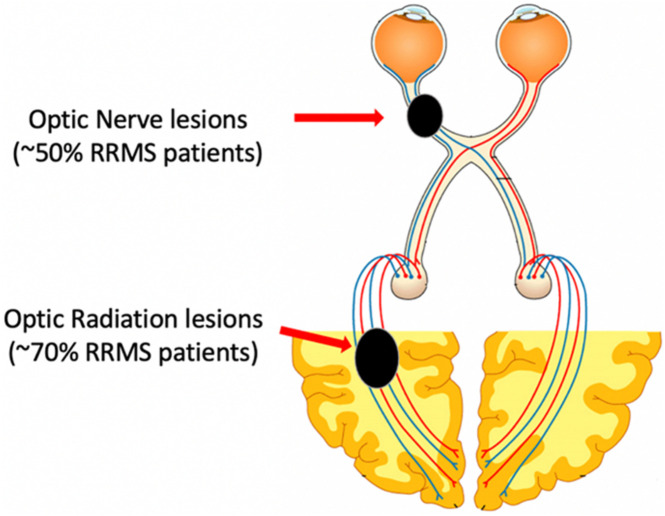
Schematic representation of the visual system and sites of MS lesions.

Secondly, the strictly hierarchical structure of the visual system provides an opportunity to follow the effect of MS damage along several levels of inter-connected neurons. Thirdly, with the advent of tractography [based on diffusion magnetic resonance imaging (MRI)], the entire length of the visual pathway, including the OR, can now be visualized and its structural damage can be quantified (Figure 2; Sherbondy et al., 2008).

**FIGURE 2:**
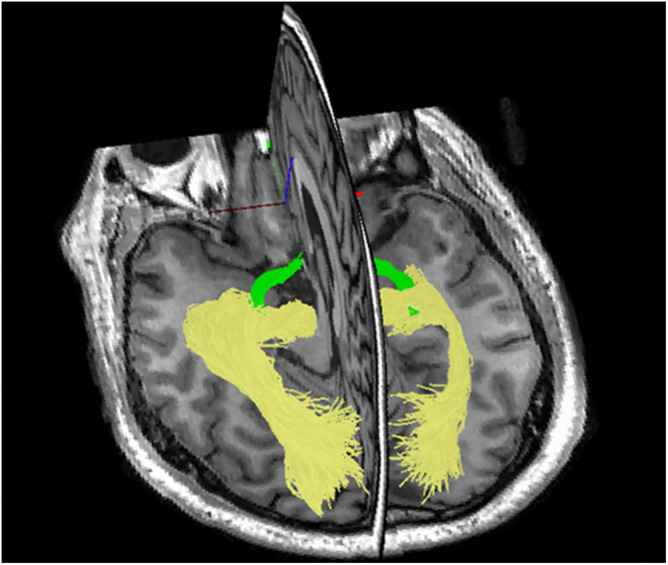
Tractography of the optic tract (green) and optic radiation using ConTrack software.

In addition, contrary to many other white matter pathways, the visual pathway allows study of the dynamics of myelin alteration in both acute and chronic lesions. This is due to the fact that, while OR lesions are typically silent, lesions of the anterior visual pathway are clinically apparent.

Furthermore, accurate and quantifiable measures of visual system function, such as visual acuity [and low contrast visual acuity (LCVA) in particular], are readily available (Balcer et al., 2017).

Finally, the visually evoked potential (VEP), an event-related potential generated by the striate cortex in response to visual stimulation (Fahle and Bach, 2006), is uniquely placed to serve as a biomarker of the myelination along the visual pathway based on the following rationale. The VEP represents an electrical signal generated at the level of striate cortex by the combined activity of post-synaptic potentials in response to visual stimulation (Creutzfeldt et al., 1969; Fahle and Bach, 2006). As a result, its magnitude (“amplitude”) and timing (“latency”) are affected by pathological changes (such, for example, as MS lesions) along the entire visual pathway. Thus, it was suggested that amplitude of the VEP reflects the number of functional fibers along the visual pathway and is determined by the severity of inflammation in the acute stage of MS lesion and subsequent axonal degeneration in later stages (Jones and Brusa, 2003). Latency, on the other hand, is related to the speed of conduction. Since the slowing of conduction affects only the demyelinated portion of the axons (Smith and Waxman, 2005; Waxman, 2005), the extent of demyelinated area is likely to be proportional to the delay of VEP arrival to the visual cortex, i.e., delay of VEP latency. As a result, in contrast to most brain lesions, the effect of myelin loss and recovery can be qualitatively measured by the latency delay (Halliday et al., 1972).

This close association between VEP latency delay and degree of visual pathway demyelination has been confirmed by clinical and experimental studies. For example, van der Walt et al. (2015) demonstrated a high degree of concordance between the length of optic nerve lesion and relative latency delay of the VEP derived from stimulation of corresponding eye (Figure 3). Similar relationships have been found in animal studies (You et al., 2011; Heidari et al., 2019). A study by Alshowaeir et al. (2014) also revealed a close relationship between VEP latency delay and lesion volume in posterior visual pathway.

**FIGURE 3:**
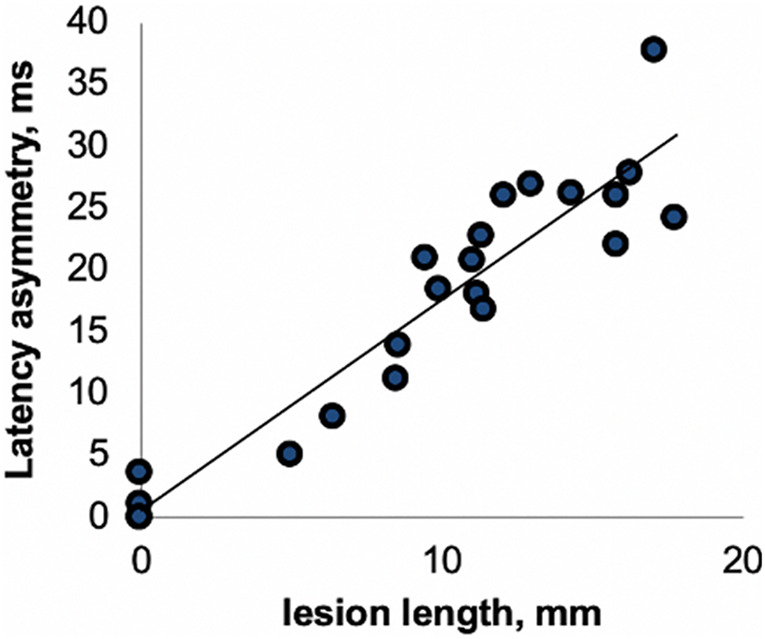
Correlation between optic nerve lesion and mfVEP latency delay [from van der Walt et al. (2015)].

Since the VEP is generated at the level of striate cortex, it is affected by pathological changes along the entire visual pathway. Therefore, latency delay of the VEP reflects the combined effect of demyelination in the entire visual system including optic nerve and OR. However, due to the unique topographic anatomy of the visual system (i.e., post-chiasmal crossing and projection of fibers subserving similar parts of the visual field of both eyes to the same area of the cortex), the effect of optic nerve and OR demyelination on VEP can be differentiated. Thus, demyelinating lesions of OR typically produce a similar delay of the VEP response in both eyes. Conversely, since lesions of the optic nerve in MS are, as a rule, unilateral, optic nerve demyelination only affects VEP recorded in response to stimulation of the affected eye. Furthermore, the monocular nature of ON allows comparison of VEP parameters recorded from the affected eye with data obtained from the fellow (unaffected) eye. Using this inter-eye latency difference (asymmetry) significantly reduces between-subject variability, providing a very accurate measure of optic nerve de/remyelination (Graham et al., 2000; Hood et al., 2000b; Klistorner et al., 2018).

There are several stimulating modalities that are employed to generate the VEP. Flash stimuli are typically used in animal studies and to record VEP response from non-cooperative patients or young children, while pattern-reversal full or half-field VEPs are commonly used in adults (Fahle and Bach, 2006). The multifocal VEP (mfVEP) represents a most recent addition to the array of VEP stimulations. The multifocal technique was initially developed by Sutter and Tran (1992); Baseler et al. (1994), Baseler and Sutter (1997) and later modified and improved to study cortical responses, first in glaucoma and later in demyelinating diseases (Klistorner et al., 1998, 2010; Hood et al., 2000a; Fraser et al., 2006).

There are several advantages of mfVEP over full-field stimulation (Table 1).

**TABLE 1:** Comparison between full-field and multifocal VEP.

	**Full-field VEP**	**Multifocal VEP**
Retinal topography of the response	Dominated by the macular region	Equally distributed within central 48° of the visual field
Number of stimulating fields	Single	Multiple (up to 56)
Number of responses in individual recording	Single combined response	Independent responses from multiple small areas of the visual field
Ability to assess retinal topography of the response	No	Yes
Susceptible to cancelation between upper and lower hemifields	Yes	No
Cortically scaled stimulation	No	Yes

The conventional full-field VEP provides a summed response of all neuronal elements stimulated and is greatly dominated by the macular region due to its cortical overrepresentation (Daniel and Whittridge, 1961). It has been estimated that 65% of the total full-field VEP response represents the central 2°of the visual field (Riggs and Wooten, 1972; Yiannikas and Walsh, 1983). A small unified check size, which is commonly used for full-field pattern stimulation, is another factor that tends to bias the central response (Harter, 1970).

In addition, being the vector sum of numerous differently oriented dipoles (caused by projection of upper and lower hemifields to oppositely oriented banks of the calcarine sulcus, which is further exacerbated by local cortical convolution), the waveform of the full-field VEP is prone to unpredictable change depending on the part of the nerve/visual field affected, leading sometimes to detection of apparent rather than real amplitude and latency change (Halliday et al., 1979; Klistorner et al., 1998). This is particularly apparent in case of OR lesions.

In contrast, the mfVEP simultaneously stimulates numerous small areas (typically 56) of the visual field using pseudorandom sequences and is able to extract individual responses from each stimulated area independently and at the same time (Sutter and Tran, 1992; Klistorner et al., 1998). This, together with cortical scaling of the stimulating areas, provides a much larger field of examination, which typically extends to 25° of eccentricity. In addition, larger check size at more peripheral locations produces an optimal mfVEP response from different parts of the visual field (Figure 4A; Balachandran et al., 2002). The introduction of orthogonally oriented bipolar recording channels straddling the inion (Klistorner and Graham, 2000) also enhanced the ability of mfVEP to detect signals from all parts of the visual field regardless of the orientation of the underlying striate cortex dipole (Figure 4B).

**FIGURE 4:**
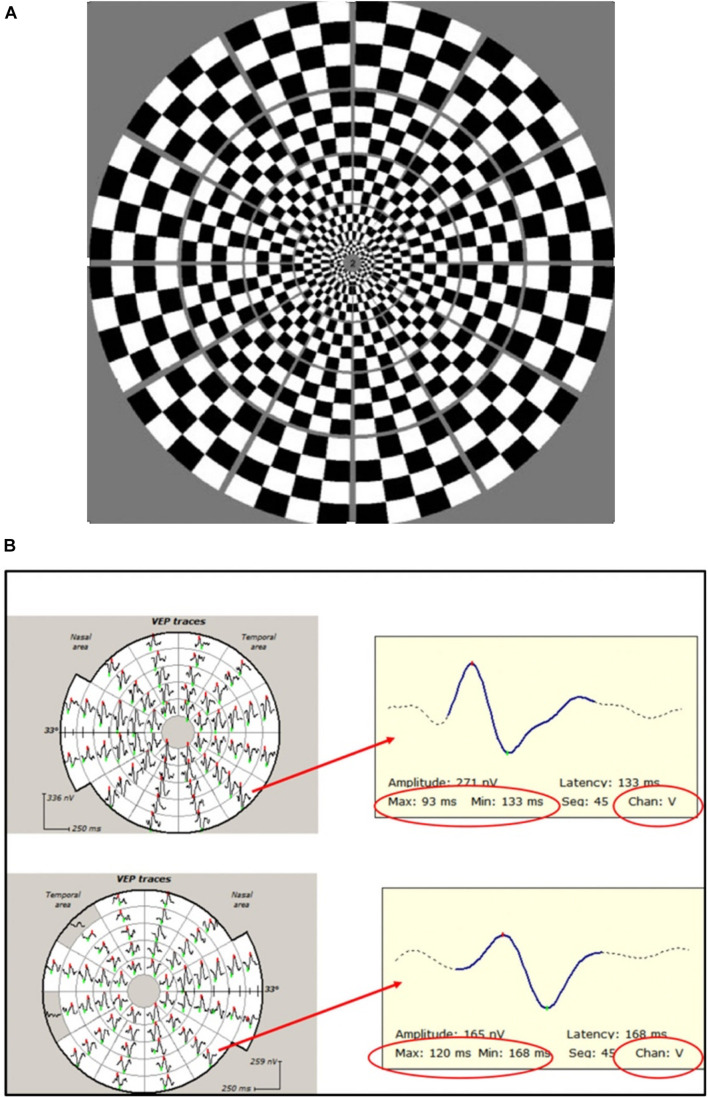
**(A)** Dartboard stimulus used in mfVEP recording. **(B)** Example of mfVEP recording from ON (bottom row) and fellow (upper row) eyes. Individual travels from corresponding segments magnified to demonstrate latency measurement. Note that the same channel (vertical) is selected for inter-eye comparison.

Furthermore, stimulation of small areas of the visual field eliminates the cancelation effect of various dipole orientation caused by the opposite position of upper and lower banks of calcarine sulcus (subserving the upper and lower hemifields) and cortical convolution, which is a serious limitation for the full-field VEP.

Since it was established that signals derived from the peripheral areas of the visual fields are less delayed and recover faster than responses derived from the central areas of the visual fields, this may also contribute to cancelation or distortion of the full-field VEP as it is a summed response (Klistorner et al., 2007).

There are various ways to measure mfVEP latency. Individual segments can be assessed independently and a retinotopically organized plot of latency delay can be constructed (both as absolute value of latency delay and deviation from a normative database) (Figure 5).

**FIGURE 5:**
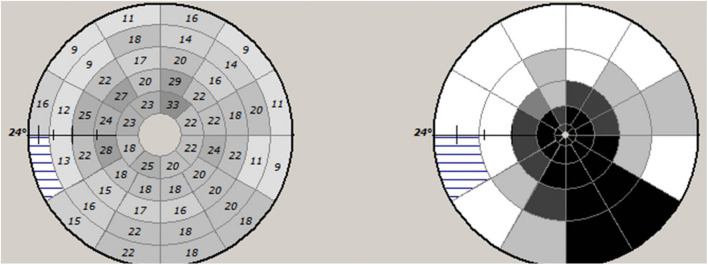
Example of mfVEP latency asymmetry values of individual segments (left) and plot of probability values of deviation from normative database.

Alternatively, an averaged value of latency across all areas of the stimulated eye can be used. However, contrary to full-field VEP, averaging of mfVEP areas does not result in cancelation or distortion of the total signal since numerical values of latency not waveforms are averaged.

In summary, the mfVEP better reflects the true state of the conductivity along the visual pathway by including information from fibers subserving more peripheral parts of the visual field and eliminating cancelation effects of differently oriented dipoles. Simultaneous recording from a plurality of visual field locations and use of orthogonal channels also results in higher spatial resolution of the mf VEP technique, allowing independent assessment of multiple regions.

While a number of imaging techniques such as magnetization transfer ratio, diffusion tensor imaging, and myelin water fraction have been recently suggested as potential biomarkers for de/remyelination in MS lesions (Jelescu et al., 2016; van der Weijden et al., 2020; Klistorner S. A. et al., 2021), there is no current consensus on an issue of which one should be selected. There are also no clinical studies to compare sensitivity and specificity between imaging and electrophysiological (and VEP in particular) techniques in assessing de/remyelination.

## Acute Lesions of the Visual Pathway

The majority of new white matter lesions in MS are clinically silent and, as a result, are typically detected during routine MRI examination long after the acute inflammatory stage. Optic nerve lesions, on the other hand, are usually clinically apparent from the onset and, therefore, provide a unique platform to study spontaneous remyelination, as well as treatment-induced myelin repair in the early post-acute period using VEP latency as a biomarker (Hood et al., 2000a; Klistorner et al., 2010; Cadavid et al., 2017; Klistorner A. et al., 2021).

Furthermore, since precise timing of ON onset is known, the ON model also offers an opportunity to investigate the effect of the lesion’s age on remyelinating capacity of any potential treating agent by studying patients with different post-ON intervals.

This is of particular importance since it is believed that remyelination is more likely to succeed in the acute or recent MS lesion, while the environment for successful remyelination may become less permissive in longstanding lesions (Chari and Blakemore, 2002; Ruffini et al., 2004). The “window of opportunity” for the process of remyelination to be successful (Blakemore et al., 2002) may be related to pro-reparative interactions between various cell populations and cytokines within the early MS lesion (Chari and Blakemore, 2002; Foote and Blakemore, 2005; Zhao et al., 2005). This critical period may open following sufficient expansion and differentiation of perilesional and lesional oligodendrocyte precursor cells and end with the conversion of acute to chronic inflammation status (Kotter et al., 2011). This age effect on the lesion is therefore likely to influence both spontaneous and treatment-induced remyelination that may be achieved.

### Multifocal Visually Evoked Potential Studies of Spontaneous Remyelination of Acute Lesions

Both experimental and clinical studies have demonstrated that after a brief block of conduction caused by acute inflammation, the surviving, but chronically demyelinated axons largely recover the ability to conduct (Smith and Waxman, 2005; Klistorner et al., 2010). This general pattern is well reflected in clinical and electrophysiological recovery after an episode of acute ON. It was shown that after the resolution of acute inflammation that typically occurs within 1–2 weeks from the onset of ON, the conduction along the demyelinated part of the affected axons resumes, resulting in restoration of vision and recovery of VEP amplitude. However, similar to full-field VEP, immediately after recovery of the conduction block, the latency of mfVEP often displays significant prolongation. We have previously demonstrated that this latency delay is highly proportional to the length of the acute demyelinated area along the optic nerve (Figure 3; Klistorner et al., 2010) and, therefore, reflects the degree of initial myelin loss (Hood et al., 2000a; Jones and Brusa, 2003; Klistorner et al., 2010; van der Walt et al., 2015).

Subsequent shortening of mfVEP latency, which is frequently observed after this initial delay, is thought to represent the process of spontaneous remyelination (Hood et al., 2000a; Klistorner et al., 2010; van der Walt et al., 2015). The mfVEP latency improvement, however, is limited in magnitude and restricted in time (Klistorner et al., 2010). Thus, the speed of latency recovery is fastest during first 3 months after an acute episode of ON, but gradually decelerates in the following months and finally ceases by the end of the first year, remaining stable thereafter (Klistorner et al., 2010, Klistorner et al., 2020).

**FIGURE 6:**
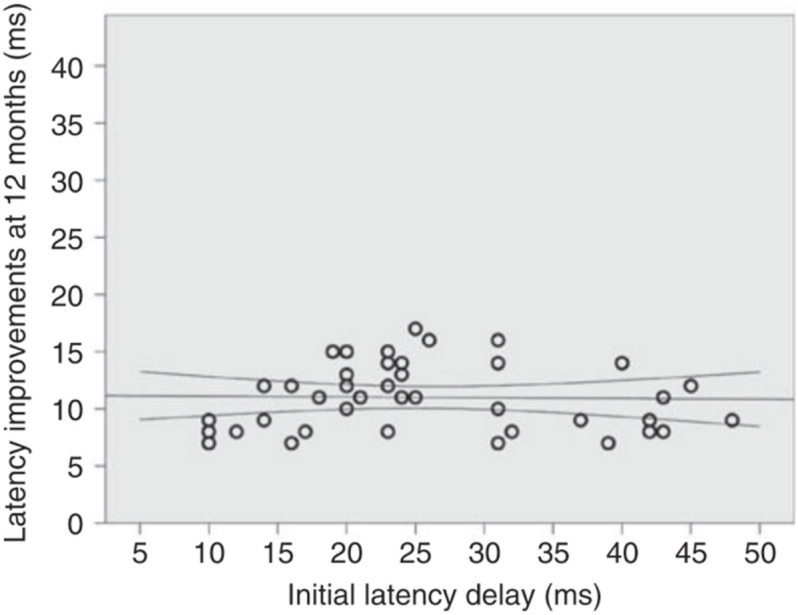
Absolute latency recovery values at 12 months plotted against baseline latency delay [from Klistorner et al. (2010)].

In addition, it was demonstrated that, at least in the optic nerve, the magnitude of post-acute latency shortening (i.e., spontaneous remyelination) is largely independent of initial latency delay (presumed size of the initial demyelinated lesion). For example, while in some cases initial (4 weeks after ON onset) latency delay of the mfVEP exceeds 35–40 ms (indicating almost total demyelination of the optic nerve), latency improvement does not usually go beyond 10–15 ms (average latency recovery 11.3 ± 3 ms) (Klistorner et al., 2010), indicating disproportionately small remyelination of large lesions (Figure 6). This partial recovery of mfVEP latency (van der Walt et al., 2015) reflects the limited nature of spontaneous remyelination, which is well documented in experimental and pathological studies (see Cunniffe and Coles, 2019 for review).

### Multifocal Visually Evoked Potential Studies of Treatment-Induced Remyelination of Acute Lesions

The acute ON model is becoming a method of choice for clinical trials aimed at myelin repair (Tsakiri et al., 2012; Galetta et al., 2015; Cadavid et al., 2017; Klistorner et al., 2018).

The mfVEP has recently been used to study remyelination in a clinical trial of monoclonal antibody opicinumab, which previously shows remyelinating activity in pre-clinical studies (RENEW and RENEWED). In the RENEW study, patients were treated with 100 mg/kg opicinumab for 20 weeks and assessed up to week 32, while the RENEWED study was designed as a follow-up study at 2 years after the last visit of RENEW study.

**FIGURE 7:**
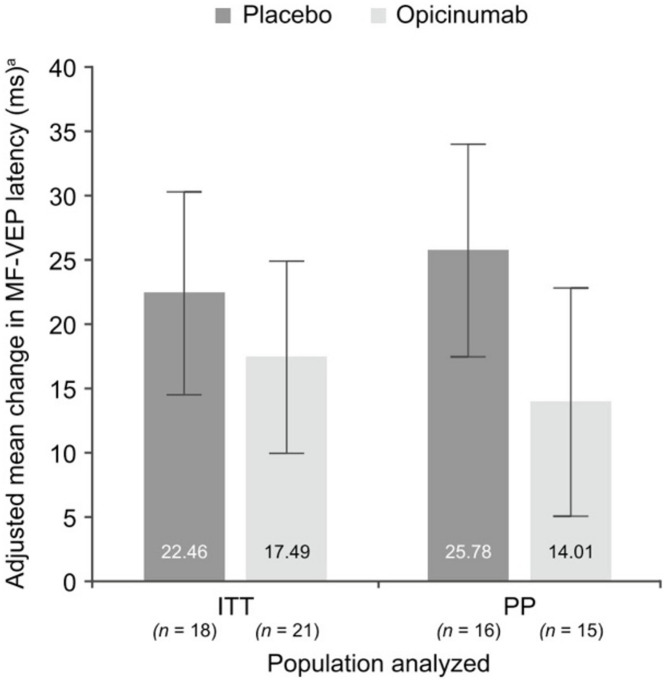
Mean change in mfVEP latency, adjusted for the baseline latency of unaffected fellow eye, at week 24 in the affected eye compared with the unaffected fellow eye at baseline in the substudy ITT and PP populations [from Klistorner et al. (2018)].

In the RENEW study, both the conventional full-field VEP (which was the primary endpoint of the study) and the mfVEP latency demonstrated a larger improvement in ON eyes of patients treated with opicinumab compared to placebo (Cadavid et al., 2017; Klistorner et al., 2018), although this only reached borderline significance. The average latency improvement in treated eyes compared to placebo was 7.6 ms in full-field VEP and 11.8 ms in mfVEP in the per-protocol population (Figure 7). The mfVEP result, however, was achieved with half of the sample size compared to full-field VEP (39 vs. 82 patients). The sample size advantage of using mfVEP was confirmed by a *post hoc* comparison of estimated effect size for change in mfVEP and full-field VEP latency for opicinumab versus placebo at week 24 in the intention-to-treat population, which showed that the mfVEP demonstrated a larger treatment effect size than full-field VEP (Klistorner et al., 2018).

Furthermore, while the high variability of full-field VEP precluded any meaningful assessment of amplitude, analysis of mfVEP demonstrated evidence that fellow eye amplitude loss occurs after ON but can potentially be prevented by opicinumab treatment (Klistorner et al., 2018).

The RENEWED study also demonstrated higher sensitivity of mfVEP in monitoring treatment-induced remyelination compared to full-field VEP. The average difference between latency recovery of mfVEP in the treated vs. placebo group increased from 14.4 to 19.6 ms over the 2 years after treatment was terminated, while full-field VEP demonstrated reduction of latency recovery from 9.4 to 6.0 ms during the same period (Aktas et al., 2020).

Further analysis of mfVEP revealed that in the opicinumab group, there was a strong association between the degree of latency delay at baseline (as measured at week 4) and the latency recovery at RENEWED day 1 (*r*^2^ = 0.72, *p* = 0.004, Pearson correlation coefficient). Conversely, the magnitude of mfVEP latency recovery was limited in the placebo group and did not correlate with initial degree of latency delay (*p* = 0.2) (Klistorner et al., 2020), which was consistent with the results of the “natural history” study of spontaneous optic nerve remyelination following an episode of acute ON reported earlier (Klistorner et al., 2010).

Therefore, in the presumed treatment-induced (opicinumab) remyelination following acute ON, the degree of myelin recovery was highly proportional to the extent of initial myelin loss (Klistorner et al., 2020).

It must be noted that while the degree of acute demyelination cannot often be assessed because of frequent incidence of edema and conduction block, continuous conduction along the demyelinated part of the affected axons typically resumes by 3–4 weeks, which still provides a good indication of the extent of original demyelination (van der Walt et al., 2015).

## Chronic Lesions of the Visual Pathway

While the clinical potential for remyelination of chronic lesions is more challenging (see discussion related to “window of opportunity” above), it is also extremely important since the diagnosis of MS is typically delayed (Klistorner et al., 2017) due to the fact that majority of MS lesions are clinically silent. As a result, it is exceedingly difficult to identify acute lesions. In addition, by the time of MS diagnosis, the patient often presents with a number of chronic brain lesions.

The visual system can also be used to monitor myelin alteration in chronic lesions. As stated above, since mfVEP is generated at the level of primary (striate) visual cortex but reflects the integrity of the full visual pathway, it is affected by the speed of conduction and, therefore, degree of de/remyelination along the entire pathway, including optic nerve and OR. Accordingly, delay of mfVEP latency in non-acute ON patients does reflect the combined effect of chronic demyelination in both optic nerve and OR. Since the effect of a chronic optic nerve lesion on mfVEP is usually monocular, while OR lesions will yield binocular latency delay due to partial chiasmal crossing of visual pathway, this provides a point of differentiation.

**FIGURE 8:**
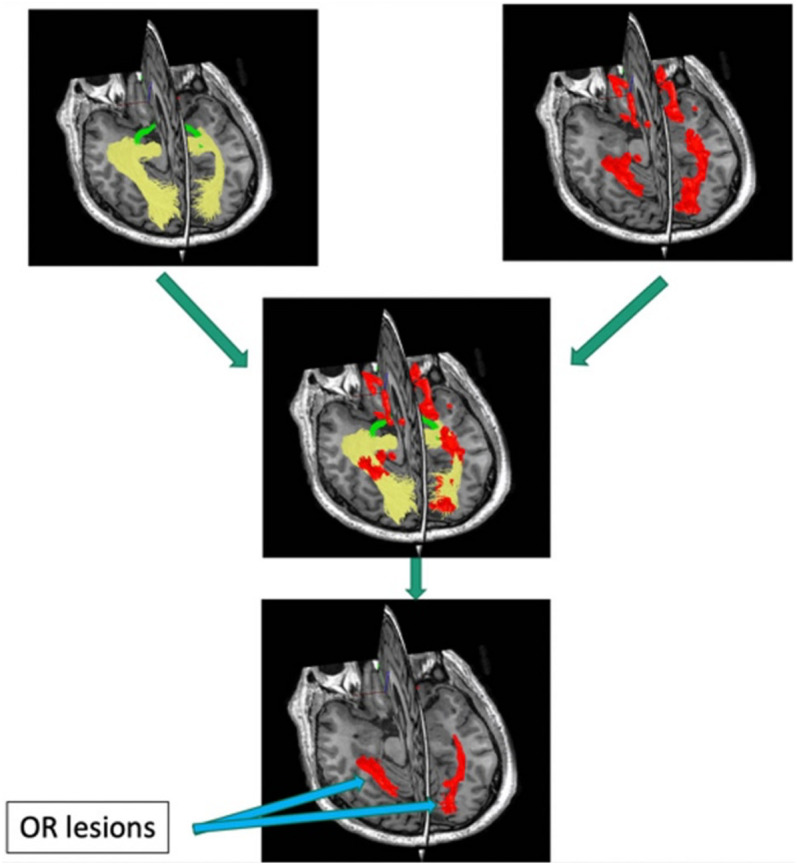
Pipeline for detecting optic radiation lesions. Optic radiation (determined by tractography in yellow) is intersected with brain lesion mask (red).

Quantitative investigation of the association between mfVEP latency delay and MS-related damage of posterior visual pathway was aided by the relatively recent development of diffusion-based tractography, which enabled identification and segmentation of major white matter tracts including ORs (Sherbondy et al., 2008). Intersection of the brain lesion mask with OR mask obtained using brain white matter tractography (Figure 8) enabled accurate volumetric assessment of the OR lesions and demonstrated significant association between structural MRI-based estimation and electrophysiological measurement of OR demyelination (Alshowaeir et al., 2014), and confirmed the above relationship between OR lesions and binocular latency delays.

### Multifocal Visually Evoked Potential Studies of Treatment-Induced Remyelination of Chronic Lesions

The mfVEP has also recently been employed as a biomarker for clinical trials to examine possible remyelination in chronic lesions, in combination with MRI. The utility of the mfVEP is further strengthened by our longitudinal analysis, which demonstrated the remarkably stable nature of mfVEP latency after 12 months in the absence of new lesional activity in the visual pathway (Klistorner et al., 2020).

While patient recruitment within a short window after symptom onset represents a limiting factor for acute ON trials, selection of patients for remyelinating trials based on chronic visual pathway lesions is less challenging. The main enrollment criteria for such trials are the presence of measurable mfVEP signal (includes ∼95% of RRMS population) and significant latency delay indicating chronic demyelination along the visual pathway (includes ∼70% of RRMS population). Furthermore, the sample size calculated for 12 months in a clinical trial of a potential remyelinating agent based on latency of the mfVEP revealed that a relatively small sample size would be required to demonstrate efficacy of remyelination therapy (Klistorner et al., 2020). This approach has been tested in a substudy of the large clinical trial of opicinumab (SYNERGY, Biogen) and is currently employed in the VISIONARY-MS trial to test the efficacy of gold nanoparticles (Clene Nanomedicine, United States) and the CCMR Two trial to test the combination of metformin and clemastine (University of Cambridge, United States) in remyelination of chronic MS lesions.

### Multifocal Visually Evoked Potential in Other Neurological Conditions

While mfVEP has also been used in monitoring other neurological conditions, such as neurofibromatosis, Leber’s optic neuropathy, chronic inflammatory demyelinating polyneuropathy, optic disc drusen, chiasmal decompression, compressive optic neuropathy, and schizophrenia (Semela et al., 2009; Yamada et al., 2011; Raz et al., 2015; Ziccardi et al., 2015; Malmqvist et al., 2017; Graf et al., 2018; Jayanetti et al., 2018), its application is sporadic and clinical usefulness is limited.

## Author Contributions

Both authors listed have made a substantial, direct and intellectual contribution to the work, and approved it for publication.

## Conflict of Interest

The authors declare that the research was conducted in the absence of any commercial or financial relationships that could be construed as a potential conflict of interest.

## Publisher’s Note

All claims expressed in this article are solely those of the authors and do not necessarily represent those of their affiliated organizations, or those of the publisher, the editors and the reviewers. Any product that may be evaluated in this article, or claim that may be made by its manufacturer, is not guaranteed or endorsed by the publisher.

## References

[B1] Aktas O. Ziemssen F. Ziemssen T. Comi G. Butzkueven H. Izquierdo G. (2020). *RENEWED: Long-Term Electrophysiological and Clinical Outcomes in Participants Previously Enrolled in the Opicinumab Phase 2 Study RENEW.* Philadelphia, PA: Wolters Kluwer Health, Inc.

[B2] Alshowaeir D. Yiannikas C. Garrick R. Parratt J. Barnett M. H. Graham S. L. (2014). Latency of multifocal visual evoked potentials in nonoptic neuritis eyes of multiple sclerosis patients associated with optic radiation lesions. *Invest. Ophthalmol. Vis. Sci.* 55 3758–3764. 10.1167/iovs.14-14571 24833744

[B3] Balachandran C. Klistorner A. Graham S. L. (2002). Effect of stimulus check size on multifocal visual evoked potentials. *Doc. Ophthalmol*. 106 183–188. 10.1023/a:1022571530152 12678283

[B4] Balcer L. J. Raynowska J. Nolan R. Galetta S. L. Kapoor R. Benedict R. (2017). Validity of low-contrast letter acuity as a visual performance outcome measure for multiple sclerosis. *Mult. Scler.* 23 734–747. 10.1177/1352458517690822 28206829 PMC5407511

[B5] Barkhof F. Calabresi P. A. Miller D. H. Reingold S. C. (2009). Imaging outcomes for neuroprotection and repair in multiple sclerosis trials. *Nat. Rev. Neurol.* 5 256–266. 10.1038/nrneurol.2009.41 19488083

[B6] Baseler H. A. Sutter E. E. (1997). M and P components of the VEP and their visual field contribution. *Vis. Res.* 37 675–790. 10.1016/S0042-6989(96)00209-X 9156212

[B7] Baseler H. A. Sutter E. E. Klein S. A. Carney T. (1994). The topography of visual evoked response properties across the visual field. *Electroencephal. Clin. Neurophysiol.* 90 65–81. 10.1016/0013-4694(94)90114-7 7509275

[B8] Blakemore W. F. Chari D. M. Gilson J. M. Crang A. J. (2002). Modelling large areas of demyelination in the rat reveals the potential and possible limitations of transplanted glial cells for remyelination in the CNS. *Glia* 38 155–168. 10.1002/glia.10067 11948809

[B9] Bramow S. Frischer J. M. Lassmann H. Koch-Henriksen N. Lucchinetti C. F. Sorensen P. S. (2010). Demyelination versus remyelination in progressive multiple sclerosis. *Brain* 133 2983–2998. 10.1093/brain/awq250 20855416

[B10] Bruck W. Kuhlmann T. Stadelmann C. (2003). Remyelination in multiple sclerosis. *J. Neurol. Sci.* 206 181–185. 10.1016/S0022-510X(02)00191-0 12559508

[B11] Cadavid D. Balcer L. Galetta S. Aktas O. Ziemssen T. Vanopdenbosch L. (2017). Safety and efficacy of opicinumab in acute optic neuritis (RENEW): a randomised, placebo-controlled, phase 2 trial. *Lancet Neurol.* 16 189–199. 10.1016/S1474-4422(16)30377-5 28229892

[B12] Chari D. M. Blakemore W. F. (2002). New insights into remyelination failure in multiple sclerosis: implications for glial cell transplantation. *Mult. Scler.* 8 271–277. 10.1191/1352458502ms842oa 12166495

[B13] Correale J. Gaitán M. I. Ysrraelit M. C. Fiol M. P. (2017). Progressive multiple sclerosis: from pathogenic mechanisms to treatment multiple sclerosis. *Brain* 140 527–546. 10.1093/brain/aww258 27794524

[B14] Creutzfeldt O. Maekawa K. Hosli L. (1969). Forms of spontaneous and evoked postsynaptic potentials of cortical nerve cells. *Prog. Brain Res.* 31 265–273. 10.1016/S0079-6123(08)63245-8 4310254

[B15] Cunniffe N. Coles A. (2019). Promoting remyelination in multiple sclerosis. *J. Neurol.* 268 30–44. 10.1007/s00415-019-09421-x 31190170 PMC7815564

[B16] Daniel P. M. Whittridge D. (1961). The representation of the visual field on the cerebral cortex in monkeys. *J. Physiol.* 159 203–221. 10.1113/jphysiol.1961.sp006803 13883391 PMC1359500

[B17] Fahle M. Bach M. (2006). “Origin of the visual evoked potentials,” in *Principles and Practice of Clinical Electrophysiology of Vision*, eds Heckenlively J. R. Arden G. B. (Cambridge: The Mit Press), 207–234.

[B18] Felts P. A. Baker T. A. Smith K. J. (1997). Conduction in segmentally demyelinated mammalian central axons. *J. Neurosci.* 17 7267–7277. 10.1523/JNEUROSCI.17-19-07267.1997 9295373 PMC6573430

[B19] Foote A. K. Blakemore W. F. (2005). Inflammation stimulates remyelination in areas of chronic demyelination. *Brain* 128 528–539. 10.1093/brain/awh417 15699059

[B20] Fraser C. Klistorner A. Graham S. L. Garrick R. Billson F. Grigg J. R. (2006). Multifocal visual evoked potential latency analysis: predicting progression to multiple sclerosis. *Arch. Neurol.* 63 847–850. 10.1001/archneur.63.6.847 16769865

[B21] Galetta S. L. Villoslada P. Levin N. Shindler K. Ishikawa H. Parr E. (2015). Acute optic neuritis: unmet clinical needs and model for new therapies. *Neurol. Neuroimmunol. Neuroinflamm.* 2:e135. 10.1212/NXI.0000000000000135 26236761 PMC4516397

[B22] Graf J. Jansen L. Ingwersen J. Ringelstein M. Harmel J. Rybak J. (2018). Multifocal visual evoked potentials in chronic inflammatory demyelinating polyneuropathy. *Ann. Clin. Trans. Neurol.* 5 952–961. 10.1002/acn3.593 30128319 PMC6093840

[B23] Graham S. L. L. Klistorner A. I. I. Grigg J. R. R. Billson F. A. (2000). Objective vep perimetry in glaucoma: asymmetry analysis to identify early deficits. *J. Glaucoma* 9 10–19. 10.1097/00061198-200002000-00004 10708226

[B24] Halliday A. M. Darbett G. Blumhardt L. D. Kriss A. (1979). “The macular and submacular subcomponents of the pattern evoked response,” in *Human Evoked Potentials*, ed. LDaG B. (New York, NY: Plenum Publishing), 135–151. 10.1007/978-1-4684-3483-5_10

[B25] Halliday A. M. McDonald W. I. Mushin J. (1972). Delayed visual evoked response in optic neuritis. *Lancet* 1 982–985. 10.1016/S0140-6736(72)91155-5 4112367

[B26] Harter M. R. (1970). Evoked cortical responses to checkerboard patterns: effect of check-size as a function of retinal eccentricity. *Vis. Res.* 10 1365–1376. 10.1016/0042-6989(70)90088-X 5516538

[B27] Heidari M. Radcliff A. B. McLellan G. S. Ver Hoeve J. N. Chan K. Kiland J. A. (2019). Evoked potentials as a biomarker of remyelination. *Proc. Natl. Acad. Sci. U.S.A.* 116 27074–27083. 10.1073/pnas.1906358116 31843913 PMC6936696

[B28] Hood D. C. Odel J. G. Zhang X. (2000a). Tracking the Recovery of Local Optic Nerve Function after Optic Neuritis:A Multifocal VEP Study. *Invest. Ophthalmol. Vis. Sci.* 41 4032–4038. 11053309

[B29] Hood D. C. Zhang X. Greenstein V. C. Kangovi S. Odel J. G. Liebmann J. M. (2000b). an interocular comparison of the multifocal VEP: a possible technique for detecting local damage to the optic nerve. *Invest. Ophthalmol. Vis. Sci.* 41 1580–1587. 10798679

[B30] Hornabrook R. S. Miller D. Newton M. R. MacManus D. G. du Boulay G. H. Halliday A. M. (1992). frequent involvement of optic radiation in patients with acute isolated optic neuritis. *Neurology* 42 77–79. 10.1212/WNL.42.1.77 1734327

[B31] Jayanetti V. Klistorner A. Graham S. L. Dexter M. Flaherty M. P. Jones K. (2018). Monitoring of optic nerve function in neurofibromatosis 2 children with optic nerve sheath meningiomas using multifocal visual evoked potentials. *J. Clin. Neurosci.* 50 262–267. 10.1016/j.jocn.2018.01.012 29398196

[B32] Jelescu I. O. Zurek M. Winters K. V. Veraart J. Rajaratnam A. Kim N. S. (2016). In vivo quantification of demyelination and recovery using compartment-specific diffusion MRI metrics validated by electron microscopy. *NeuroImage* 132 104–114. 10.1016/j.neuroimage.2016.02.004 26876473 PMC4851889

[B33] Jenkins T. Ciccarelli O. Toosy A. Miszkiel K. Wheeler-Kingshott C. Altmann D. (2010). Dissecting structure-function interactions in acute optic neuritis to investigate neuroplasticity. *Hum. Brain Map.* 31 276–286. 10.1002/hbm.20863 19662659 PMC6870769

[B34] Jones S. J. Brusa A. (2003). Neurophysiological evidence for long-term repair of MS lesions: implications for axon protection. *J. Neurol. Sci.* 206 193–198. 10.1016/S0022-510X(02)00428-8 12559510

[B35] Klistorner A. I. I. Graham S. L. L. Grigg J. R. R. Billson F. A. (1998). Multifocal topographic visual evoked potential: improving objective detection of local visual field defects. *Invest. Ophthalmol. Vis. Sci.* 39 937–950. 9579473

[B36] Klistorner A. Graham S. L. L. (2000). Objective perimetry in glaucoma. *Ophthalmology* 107 2283–2299. 10.1016/S0161-6420(00)00367-5 11097611

[B37] Klistorner A. Arvind H. Garrick R. Yiannikas C. Paine M. Graham S. L. (2010). Remyelination of optic nerve lesions: spatial and temporal factors. *Mult. Scler.* 16 786–795. 10.1177/1352458510371408 20530125

[B38] Klistorner A. Chai Y. Leocani L. Albrecht P. Aktas O. Butzkueven H. (2018). Assessment of opicinumab in acute optic neuritis using multifocal visual evoked potential. *CNS Drugs* 32 1159–1171. 10.1007/s40263-018-0575-8 30267385 PMC6280853

[B39] Klistorner A. Graham E. C. Yiannikas C. Barnett M. Parratt J. Garrick R. (2017). Progression of Retinal Ganglion Cell Loss in Multiple Sclerosis Is Associated with New Lesions in the Optic Radiations. *Eur. J. Neurol.* 24 1392–1398. 10.1111/ene.13404 28799222

[B40] Klistorner A. Graham S. Fraser C. Garrick R. Nguyen T. Paine M. (2007). Electrophysiological evidence for heterogeneity of lesions in optic neuritis. *Invest Ophthalmol. Vis. Sci.* 48 4549–4556. 10.1167/iovs.07-0381 17898277

[B41] Klistorner A. Naylor M. Zhu B. (2020). RENEWED: long-term mfvep latency outcomes in participants previously enrolled in the opicinumab phase 2 study RENEW. *Mult. Scler. J.* 26 492. 10.1177/1352458520974937

[B42] Klistorner A. Triplett J. D. Barnett M. H. Yiannikas C. Barton J. Parratt J. (2021). Latency of multifocal visual evoked potential in multiple sclerosis. *J. Clin. Neurophysiol*. 38, 186–191. 33235179 10.1097/WNP.0000000000000757

[B43] Klistorner A. Vootakuru N. Wang C. Yiannikas C. Graham S. L. Parratt J. (2015). Decoding diffusivity in multiple sclerosis: analysis of optic radiation lesional and non-lesional white matter. *PLoS One* 10:e0122114. 10.1371/journal.pone.0122114 25807541 PMC4373765

[B44] Klistorner S. A. Barnett M. H. Wasserthal J. Yiannikas C. Barton J. Parratt J. (2021). Differentiating axonal loss and demyelination in chronic MS lesions: a novel approach using single streamline diffusivity analysis. *PLoS One* 16:e0244776. 10.1371/journal.pone.0244766 33406139 PMC7787472

[B45] Kornek B. Storch M. K. Weissert R. Wallstroem E. Stefferl A. Olsson T. (2000). Multiple sclerosis and chronic autoimmune encephalomyelitis: a comparative study of axonal injury in active, inactive and remyelinated lesons. *Am. J. Pathol.* 157 267–276. 10.1016/S0002-9440(10)64537-3 10880396 PMC1850217

[B46] Kotter M. R. Stadelmann C. Hartung H. P. (2011). Enhancing remyelination in disease–can we wrap it up? *Brain* 134(Pt 7), 1882–1900. 10.1093/brain/awr014 21507994

[B47] Lubetzki C. Zalc B. Williams A. Stadelmann C. Stankoff B. (2020). Remyelination in multiple sclerosis: from basic science to clinical translation. *Lancet Neurol.* 19 678–688. 10.1016/S1474-4422(20)30140-X 32702337

[B48] Mallik S. Samson R. S. Wheeler-Kingshott C. A. Miller D. H. (2014). Imaging outcomes for trials of remyelination in multiple sclerosis. *J. Neurol. Neurosurg. Psychiatry* 85 1396–1404. 10.1136/jnnp-2014-307650 24769473 PMC4335693

[B49] Malmqvist L. de Santiago L. Boquete L. Hamann S. (2017). Multifocal visual evoked potentials for quantifying optic nerve dysfunction in patients with optic disc drusen. *Acta Ophthalmol.* 95 357–362. 10.1111/aos.13347 28139892

[B50] Oh J. Ontaneda D. Azevedo C. Klawiter E. C. Absinta M. Arnold D. L. (2019). Imaging outcome measures of neuroprotection and repair in MS: a consensus statement from NAIMS. *Neurology* 12 519–533. 10.1212/WNL.0000000000007099 30787160 PMC6511106

[B51] Patrikios P. Stadelmann C. Kutzelnigg A. Rauschka H. Schmidbauer M. Laursen H. (2006). remyelination is extensive in a subset of multiple sclerosis patients. *Brain* 129 3165–3172. 10.1093/brain/awl217 16921173

[B52] Périer O. Grégoire A. (1965). Electron microscopic features of multiple sclerosis lesions. *Brain* 88 937–952. 10.1093/brain/88.5.937 5864468

[B53] Peterson L. K. Fujinami R. S. (2007). Inflammation, demyelination, neurodegeneration and neuroprotectionin the pathogenesis of multiple sclerosis. *J. Neuroimunol.* 184 37–44. 10.1016/j.jneuroim.2006.11.015 17196667 PMC1933528

[B54] Prineas J. W. Kwon E. E. Goldenberg P. Z. Ilyas A. A. Quarles R. H. Benjamins J. A. (1989). Multiple sclerosis. oligodendrocyte proliferation and differentiation in fresh lesions. *Lab. Invest.* 61 489–503. 2811298

[B55] Raine C. S. Wu E. (1993). Multiple sclerosis: remyelination in acute lesions. *J. Neuropathol. Exp. Neurol.* 52 199–204. 10.1097/00005072-199305000-00003 7684075

[B56] Raz N. Bick A. S. Klistorner A. Spektor S. Reich D. S. Ben-Hur T. (2015). Physiological correlates and predictors of functional recovery after chiasmal decompression. *J. Neuroophthalmol.* 35 348–352. 10.1097/WNO.0000000000000266 25996300 PMC5078717

[B57] Riggs L. A. Wooten B. R. (1972). “Electrical measures and psychophysical data on human vision,” in *Handbook of Sensory Physiology*, eds Jameson D. Hurvich L. M. (New York, NY: Springer-Verlag), 690–731. 10.1007/978-3-642-88658-4_27

[B58] Ruffini F. Kennedy T. E. Antel J. P. (2004). Inflammation and remyelination in the central nervous system. *Am. J. Pathol.* 164 1519–1522. 10.1016/S0002-9440(10)63709-1 15111297 PMC1615648

[B59] Semela L. Yang E. B. THedges R. Vuong L. Odel J. G. Hood D. C. (2009). Multifocal visual-evoked potential in unilateral compressive optic neuropathy. *Invest. Ophtal. Vis. Sci.* 50 4199–4204. 10.1136/bjo.2006.097980 PMC1994728 17077118

[B60] Sherbondy A. J. Dougherty R. F. Napel S. Wandell B. A. (2008). Identifying the human optic radiation using diffusion imaging and fiber tractography. *J. Vis.* 8 12.1–1211. 10.1167/8.10.12 PMC2759943 19146354

[B61] Smith K. J. Waxman S. G. (2005). “The conduction properties of demyelinated and remyelinated axons,” in *Multiple Sclerosis as Neuronal Disease*, ed. Waxman S. G. (Amsterdam: Elsevier Academic Press), 85–100. 10.1016/B978-012738761-1/50007-9

[B62] Suhs K. W. Hein K. Sattler M. B. Gorlitz A. Ciupka C. Scholz K. (2012). A randomized, double-blind, phase 2 study of erythropoietin in optic neuritis. *Ann. Neurol.* 72 199–210. 10.1002/ana.23573 22926853

[B63] Sutter E. E. Tran D. (1992). The field topography of ERG components in man - 1. The photopic luminance response. *Vis. Res.* 32 433–446. 10.1016/0042-6989(92)90235-B 1604830

[B64] Trapp B. D. Ransohoff R. Fisher E. Rudick R. (1999). Neurodegeneration in multiple sclerosis: relationship to neurological disability. *Neuroscientist* 5 48–57. 10.1177/107385849900500107

[B65] Tsakiri A. Kallenbach K. Fuglø D. Wanscher B. Larsson H. Frederiksen J. (2012). Simvastatin improves final visual outcome in acute optic neuritis: a randomized study. *Mult. Scler.* 18 72–81. 10.1177/1352458511415452 21921071

[B66] van der Walt A. Kolbe S. Mitchell P. Wang Y. Butzkueven H. Egan G. (2015). Parallel changes in structural and functional measures of optic nerve myelination after optic neuritis. *PLoS One* 10:e0121084. 10.1371/journal.pone.0121084 26020925 PMC4447428

[B67] van der Weijden C. W. J. García D. V. Borra R. J. H. Thurner P. Meilof J. F. van Laar P. J. (2020). Myelin quantification with MRI: a systematic review of accuracy and reproducibility. *NeuroImage* 226:117561. 10.1016/j.neuroimage.2020.117561 33189927

[B68] Waxman S. G. (2005). “Altered distributions and functions of multiple sodium channel subtypes in multiple sclerosis and its models,” in *Multiple Sclerosis as a Neuronal Disease*, ed. Waxman S. G. (Amsterdam: Elsevier). 10.1016/B978-012738761-1/50008-0

[B69] Yamada M. Yukawa E. Yakerani F. Matsuura T. Hara Y. (2011). Multifocal visual-evoked potentials in patients with schizophrenia during treatment. *Acta Neuropsychiatr.* 23 31–34. 10.1111/j.1601-5215.2010.00509.x 25379694

[B70] Yiannikas C. Walsh J. C. (1983). The variation of the pattern shift visual evoked response with the size of the stimulus field. *Electroencephalogr. Clin. Neurophysiol.* 55 427–435. 10.1016/0013-4694(83)90131-1 6187536

[B71] You Y. Klistorner A. Thie J. Graham S. L. (2011). Latency delay of visual evoked potential is a real measurement of demyelination in a rat model of optic neuritis. *Invest. Ophthalmol. Vis. Sci.* 52 6911–6918. 10.1167/iovs.11-7434 21791585

[B72] Zhao C. Fancy S. Kotter M. R. Li W. Y. Franklin R. (2005). Mechanisms of CNS remyelination-the key to therapeutic advances. *J. Neurol. Sci.* 233 87–91. 10.1016/j.jns.2005.03.008 15949498

[B73] Ziccardi L. Parisi V. Giannini D. Sadun F. De Negri A. M. Barboni P. (2015). Multifocal VEP provide electrophysiological evidence of predominant dysfunction of the optic nerve fibers derived from the central retina in leber’s hereditary optic neuropathy. *Graefes. Arch. Clin. Exp. Ophthalmol.* 253 1591–2015. 10.1007/s00417-015-2979-1 25773998

